# Effect of Triptolide on Dextran Sodium Sulfate-Induced Ulcerative Colitis and Gut Microbiota in Mice

**DOI:** 10.3389/fphar.2019.01652

**Published:** 2020-01-29

**Authors:** Hao Wu, Quan Rao, Guang-Chao Ma, Xiao-Hong Yu, Cong-En Zhang, Zhi-Jie Ma

**Affiliations:** ^1^ Beijing Friendship Hospital, Capital Medical University, Beijing, China; ^2^ Department of General Surgery, Beijing Friendship Hospital, Capital Medical University, Beijing, China

**Keywords:** ulcerative colitis, triptolide, gut microbiota, high-throughput sequencing, mice model

## Abstract

Triptolide is beneficial for the treatment of ulcerative colitis (UC), which is closely related to the gut microbiota. However, whether the therapeutic effects of triptolide involve the regulation of the gut microbiota is still unclear. In the present study, animal models of UC mice induced by dextran sodium sulfate (DSS) were established, the changes of gut microbiota in mice were detected by high-throughput sequencing. The effects of triptolide on DSS-induced UC mouse and its gut microbiota were studied. As a result, we found that triptolide exerted anti-inflammatory and therapeutic effects on UC mice. Sequencing results for the gut microbiota showed that the composition of the gut microbiota from DSS group was disordered as compared with that from the control group, consistent with a decrease in the abundance of flora. Triptolide treatment accelerated the recovery of the population of the gut microbiota and significantly improved the microbial diversity. At the phylum level, the population of *Bacteroidetes* decreased and that of *Firmicutes* increased. At the genus level, *Bacteroides* and *Lachnospiraceae* counts decreased. Thus, triptolide could regulate the composition of the gut microbiota, accelerate the recovery of microbiota, and exert good therapeutic effects in UC mice. Our results also revealed that fecal transplantation from triptolide-treated mice could relieve UC. This study provides a reference for the rational use of triptolide for the treatment of UC.

## Introduction

Ulcerative colitis (UC) is a type of inflammatory bowel disease (IBD) characterized with chronic and repeated episodes of enteropatia. Patients with UC present with abdominal pain and bloody diarrhea, and the disease has a long course. In addition, recurrence is common along with severe complications and poor prognosis (Parkes and Jewell, 2001; Halpin and Ford, 2012; Giuffrida et al., 2018). The annual incidence of UC has been increasing, especially in developed countries (Prideaux et al., 2012). The etiology and pathogenesis of UC are incompletely understood. The constant improvement in the understanding of UC has shown that environmental factors, genetic factors, gut microbiota, and immune factors are closely related to the occurrence of UC. Therefore, this disease is not caused by a single factor but through the combination of multiple factors (Hisamatsu et al., 2013).

The interaction between the host immune system and gut microbiota is thought to be the main cause of colonic inflammation (Borody and Campbell, 2011; Ordás et al., 2012). The population of some pathogenic bacteria such as *Escherichia coli*, *Bacteroides fragilis*, and *Helicobacter* thriving in the intestinal tract of patients with UC is significantly higher than that in healthy individuals and may be related to the incidence of UC (Jess et al., 2011; Tuci et al., 2011). A correlation has been observed between UC and gut microbiota. The normal gut microbiota constitutes the intestinal mucosal barrier of the body and protects the intestinal tract. Any disturbance in the gut microbiota population may cause colonic inflammation (Strober et al., 2007).

Triptolide is a pentacyclic diterpenoid purified from *Tripterygium wilfordii*, a traditional Chinese medicine. It exerts anti-inflammatory, immunosuppressive, and antitumor activities and has been widely used for the treatment of various inflammatory diseases (Lin et al., 2001; Lee et al., 2012; Wu et al., 2013; Zheng et al., 2013; Banerjee et al., 2013). Recent studies have shown that triptolide exerts beneficial effects against UC (Wei et al., 2008; Zhang et al., 2016; Zhang and Chen, 2017). Triptolide is also closely related to the gut microbiota through its immunosuppressive effects (Goldszmid and Trinchieri, 2012; Furusawa et al., 2013). To date, only a few studies have described the effects of triptolide on the gut microbiota of a dextran sodium sulfate (DSS)-induced colitis model.

Here, we investigated whether triptolide modulates the composition of the gut microbiota and inhibits the production of inflammatory factors in a DSS-induced colitis model. Furthermore, we evaluated the therapeutic effects of triptolide on DSS-induced UC mice as well as its regulatory action on the gut microbiota. Fecal transplantations were used to further confirm that triptolide could improve the UC mice by regulating the gut microbiota. The optimum dose and toxicity of triptolide were determined and its immunomodulatory effects on DSS-induced UC mice were investigated, which provided a reference for the rational use of triptolide for UC treatment.

## Materials and Methods

### Experimental Animals

C57BL/6 male mice weighing approximately 20–22 g were purchased from the Laboratory Animal Center of the Capital Medical University (Certification number SCXK-JING 2016-0002). All animals were raised in accordance with the National Institutes of Health Guide for Laboratory animals. The study was approved by the Animal Ethics Committee of Capital Medical University. Animals were housed under standard environment conditions at 20–25°C and 12 h dark/light cycle and had free access to sterilized water and standard food. All animals were acclimated for 7 days prior to experimentation.

### Instruments and Reagents

Power Pac 3000 electrophoresis apparatus (Bio-Rad, USA), IX71 inverted microscope (Olympus, Japan), Gradient A10 Mill-Q ultrapure water (Millipore, USA), Forma-86CULT Freezer ultra-low temperature refrigerator (Thermo electron), Extraction cleaner (KQ5200E), automatic biochemical analyzer (Mindray BS-300), microplate reader (Thermo Fisher Scientific), polymerase chain reaction (PCR) instrument (ABI GeneAmp^®^ 9700, ABI, USA), ultra-microspectrophotometer (Nano Drop 2000, Thermo Fisher Scientific), Sartorius BSA224S-CW microanalytical balance (d = 0.1 mg), and refrigerated centrifuge (Sigma, Germany) were used.

Triptolide (China Food and Drug Control Institute, 111567-201404) and DSS were purchased from MP Biomedicals (MW; 36,000–50,000; MP Biomedicals, Solon, OH, USA). Enzyme-linked immunosorbent assay (ELISA) kits for tumor necrosis factor (TNF)-α, interleukin (IL)-6, and IL-17 were supplied by Thermo Fisher Scientific; aspartate aminotransferase assay kit, alanine aminotransaminase test kit, and mesalazine (enteric-coated) tablets were procured from Losan Pharma GmbH (Germany). In addition, TransStart FastPfu DNA Polymerase (AP221-02, Beijing Quanjin Biotechnology Co., Ltd.), DNA extraction kit (Omega Bio-Tek, US), and Illumina MiSeq platform (Illumina MiSeq PE300, Illumina, USA) were obtained.

### Induction of Experimental Ulcerative Colitis

A total of 60 male mice were included in this experiment. Male mice (n = 60) were randomly divided into six groups (n = 10/group). After 1 week of adaptive feeding, the mice from the control group (Con) and DSS group (Mod) were administered normal saline. The mice from positive control group (Mes) received mesalazine (50 mgċkg^−1^ċday^−1^), or the mice received triptolide at high [0.4 mg/kg, Triptolide (TP)1 group], medium (0.2 mg/kg, TP2 group), or low (0.1 mg/kg, TP3 group) dose (Qianqian et al., 2016). After 3 days, the drinking water for all groups except the Con group was supplemented with 3% DSS for 7 days (the average daily drinking water consumption per mouse is about 4.3–4.5 ml). The experimental period was 10 days.

### Fecal Microbiota Transplantation

To determine whether the gut microbiota of triptolide-treated animals may improve the condition of UC mice, we transferred the microbiota of triptolide-treated mice to UC mice. Donor mice were randomly divided into three groups (n = 6 per group) including CON, MOD, and TP. The CON group was fed normally; the MOD and TP groups received 3% DSS in drinking water to induce UC. The TP group received triptolide treatment (0.4 mg/kg) orally once per day whereas the CON group administered normal saline treatment. The stools were collected from donor mice daily in a laminar flow fume hood under aseptic conditions for 7 days. Stools of donor mice in each group were collected, and 100 mg of stools were re-suspended in 1 ml of sterile saline. The solution was vigorously mixed for 10 s with a desktop vortex and centrifuged at 800 g for 3 min. Collect the supernatant as described below and use it as graft material. Fresh graft materials were prepared within 10 min before tube feeding on the day of transplantation to prevent changes in bacterial composition. The recipient mice were randomly divided into three groups (n = 7 per group) including fecal transplantation (FT)-Con, FT-Mod, and FT-TP. Each group of recipient mice received 3% DSS in drinking to induce UC and 100 ml of fresh transplant material from the donor groups orally per day for 7 days (Borody et al., 2013; Cui et al., 2018).

### Assessment of Ulcerative Colitis

#### Disease Activity Index

During the experimental period, weights of mice were daily recorded and expressed as the percentage body weight prior to the induction of colitis. Disease activity index (DAI) was calculated from the percentage loss in body weight in combination with stool consistency and feces bleeding as follows: DAI = (weight loss score + fecal trait score + blood test score)/3) (Murano et al., 2010).

#### General Morphology Score

On day 10 of the experiment, cervical dislocation method was used to euthanize all animals. The colon was excised and its length and thickness were measured with a ruler, and relevant statistical analyses were performed. Colonic lesions were observed, and the whole colon tissue was separated; the colon was longitudinally cut, washed several times with pre-cooled saline, filtered through a filter paper, spread and fixed, and visually observed for colonic mucosal inflammation and ulceration. Scoring criteria was as follows: no damage to mucosa (0 points), mucosal congestion (1 point), ulcer in less than 25% of the damaged area (2 points), ulcer in 25–50% of the damaged area (3 points), ulcer in more than 50% of the damaged area (4 points) (Ekstrom, 1998).

#### Histological Examination

The colons from mice of all groups were excised, washed with physiological saline, fixed in 4% paraformaldehyde for 24 h, washed with water, dehydrated with ethanol, and embedded in paraffin.

Tissues were cut into standard sections and stained with hematoxylin and eosin (H&E) for histological examination. Based on the morphology of epithelial cells, the infiltration of inflammatory cells was observed by two double-blinded pathologists, and the results were averaged for scoring. Colonic histopathology score was used as the main criterion for the evaluation of the degree of inflammation and described as follows: 0 point, normal and no inflammatory cell infiltration; 1 point, mild inflammatory cell infiltration but no damage to submucosal tissues; 2 points, moderate inflammatory cell infiltration and submucosal tissue destruction (injury range 10 to 25%); 3 points, obvious inflammatory cell infiltration, submucosal tissue destruction, thickening of colon wall (injury range 25 to 50%); 4 points, severe inflammatory cell infiltration, severe colon tissue damage (>50% lesion range), and thickening of colon wall (Boirivant et al., 2001).

### Blood Collection and Measurement of Cytokines

Blood samples were directly collected from the orbit, and transferred to 2 ml Eppendorf tubes, followed by centrifugation at 1,000 g for 10 min at 4°C. The samples were stored at −80°C for biochemical assays. The levels of TNF-α, IL-6, and IL-17 in the serum were measured using a commercially available ELISA kit according to the manufacturer's instructions.

### Fecal Sample Collection and Processing

Mice were sacrificed on day 10 of the experiment, and their abdominal cavities were dissected. Complete colon tissues from anus to end of cecum were obtained and placed on an ice tray. The tissues were longitudinally dissected along the mesentery, and the fecal particles (2–3 capsules) in the colon were immediately collected and stored in liquid nitrogen. The DNA of the bacteria from the feces was extracted.

### Extraction and Sequencing of Bacterial Deoxyribonucleic Acid in Feces

#### Deoxyribonucleic Acid Extraction and Detection

The total DNA in the feces was extracted using a DNA extraction kit, and its concentration and purity were measured using NanoDrop 2000. The quality of the extracted DNA was analyzed with 1% agarose gel electrophoresis.

### Polymerase Chain Reaction Amplification and Product Recovery

The V3–V4 variable region sequence of the bacterial 16S ribosomal RNA (rRNA) gene was used as the target, while the 338F-806R with a barcode sequence was used as the primer to carry out PCR amplification. The PCR product was recovered with 2% agarose gel using AxyPrep. Purification was carried out with a DNA Gel Extraction Kit (Axygen Biosciences, Union City, CA, USA), and elution was performed with Tris hydrochloride (Tris-HCl). Detection was carried out with 2% agarose electrophoresis.

### Fluorescence Quantification

The quantitative evaluation of electrophoresis results was carried out using QuantiFluor™-ST (Promega, USA). Then according to the sequencing requirements of each sample, the corresponding proportion of mixing.

### Illumina MiSeq PE300 Sequencing

After quantitation and library construction, PCR products were subjected to high-throughput sequencing using the Illumina MiSeq PE300 platform to obtain the V3–V4 variable region base sequence information of the bacterial 16S rRNA gene. The QIUME2.0 software package was used for operational taxonomic unit (OTU) analysis for sequencing fragments. OTU clustering is an alignment analysis of the sequence with the SILVA database, and provides taxonomic units corresponding to OTU (including the phylogenetic species) and its abundance. It calculates the metric index of Chao1, Shannon and evaluates the richness and uniformity of bacterial flora in the sample. The PICRUSt software was used to predict the metabolic functions of the bacteria in each group, and the different components were evaluated and used as the reference for the subsequent function and mechanism analyses.

### Statistical Analysis

Data were analyzed using the SPSS software program (version 22.0, Chicago, IL, USA). All results are expressed as mean ± standard error of mean (SEM). One-way analysis of variance (ANOVA) was used with *post-hoc* test, followed by a test to assess significant differences in the results (the difference was considered statistically significant at *P* < 0.05 and highly significant at *P* < 0.01).

## Results

### Triptolide Improves the General Health and Survival of Mice Treated With Dextran Sodium Sulfate

**Figure 1:**
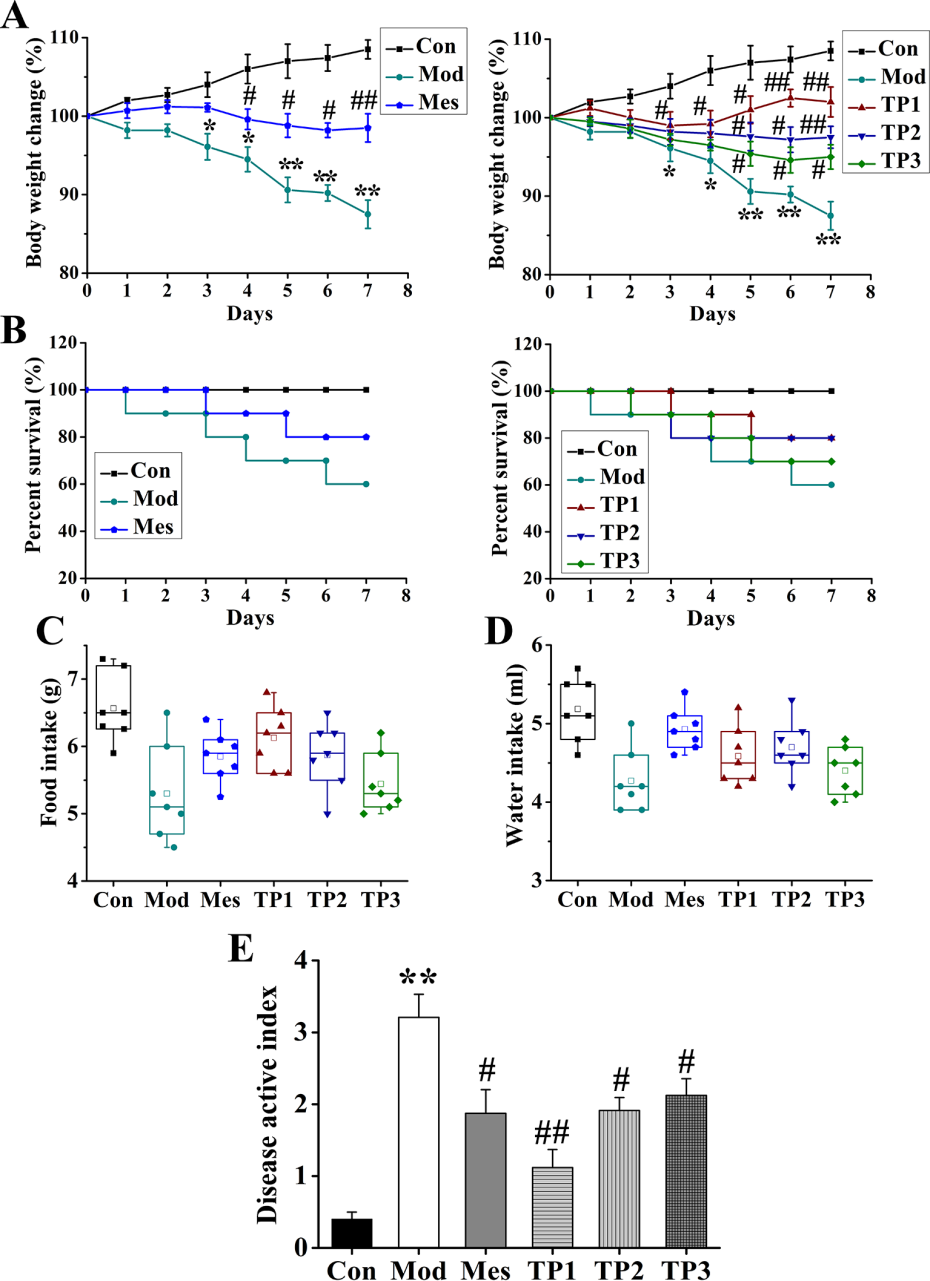
Triptolide ameliorated the effects of dextran sodium sulfate (DSS)-induced chronic ulcerative colitis (UC) in mice. **(A, B)** Body weight loss and lower survival rate were reduced after triptolide treatment in DSS-induced UC model mice. **(C, D)** Triptolide treatment increased food and water intake compared with DSS-induced UC model mice. **(E)** Mice treated with triptolide had a lower disease active index score compared with DSS-induced UC model mice. *P < 0.05 and **P < 0.01 *versus* Con group; ^#^ *P* < 0.05 and ^##^ *P* < 0.01 *versus* Mod group.

The daily weight, food intake, and water consumption of mice from each group were recorded and analyzed. The Con group had normal food and drinking water intake, and their body weight showed an upward trend. The mice from the Mod group showed a decrease in these parameters after the inception of the colitis. Their activity and body weight decreased, and the coat color was dull. The body mass of mice from TP1 group improved during the modeling period (**Figure 1A**). The general observations of stool, body mass, hair, diet, and activity were normal in Con group. Death was reported in Mod and other drug-administered groups (**Figure 1B**). During the experiment, some changes in diet and water intake were noted. The mice from Con group had the highest diet and water intake, while the lowest intake of food and water was observed for the mice from Mod group. The food and water intake were restored for the mice from drug-treated groups (**Figures 1C, D**); statistical analysis showed that compared with the Con group, the DAI score of the Mod group was significantly higher (*P* < 0.01). A significant decrease in DAI score was observed for the mice from Mes group, TP2 group, and TP3 group as compared with those from the Mod group (*P* < 0.05). The DAI score was significantly lower for the mice from TP1 group than those from the Mod group (*P* < 0.01) (**Figure 1E**).

### General Colonic and Histopathological Observations

#### Colon Length and Thickness

**Figure 2:**
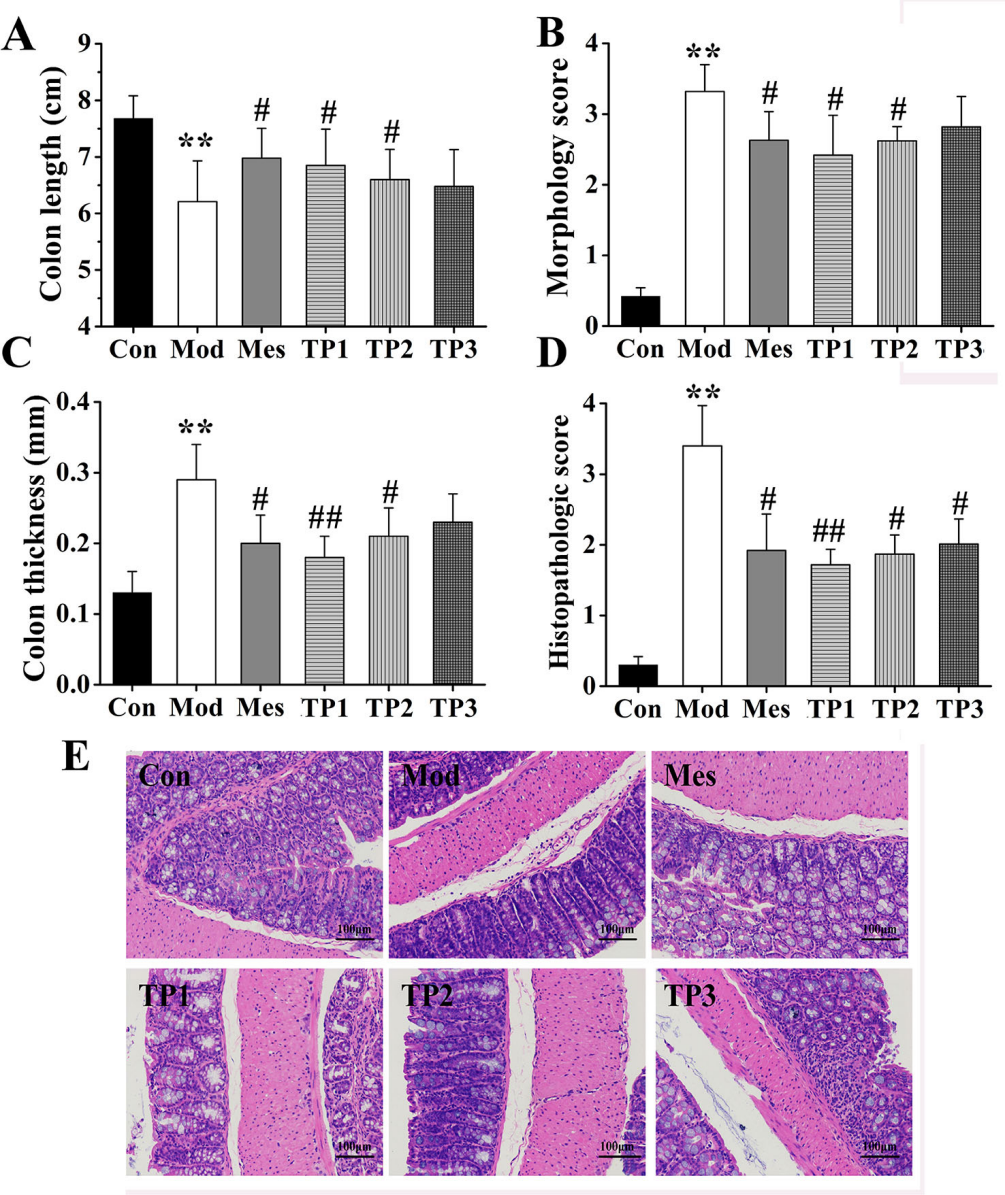
Triptolide ameliorated colonic injury in dextran sodium sulfate (DSS)-induced chronic colitis mice. **(A–C)** Mice treated with triptolide had a significantly increased colon length, decreased general morphology score, and colon thickness as compared with DSS-induced ulcerative colitis (UC) model mice. **(D, E)** hematoxylin and eosin (H&E) staining and histological colitis score showed that triptolide reduced mucosal necrosis and inflammatory cell infiltration in colon. **P < 0.01 *versus* Con group; ^#^ *P* < 0.05 and ^##^ *P* < 0.01 *versus* Mod group.

The whole colon tissue was isolated, and its length and thickness were measured with a ruler; relevant statistical analysis was performed. The results showed that the colon was the longest in the mice from the Con group and shortest in the mice from the Mod group; the difference between the colon length of the mice from Mod and Con groups was statistically significant (*P* < 0.01). The colon lengths of mice from Mes, TP1, and TP2 groups were longer than those of mice from the Mod group, and a significant difference (*P* < 0.05) was observed (**Figure 2A**). The comparison of colon thickness revealed maximum thickness for the samples from the Mod group, which may be related to the serious colonic injury. Colon thickness significantly increased in the mice from Mod group as compared with that for mice from the Con group (*P* < 0.01). The colon thickness for Mes and TP2 groups was significantly lower than that for the Mod group (*P* < 0.05), while that for the mice from TP1 group was significantly lower than the colon thickness of mice from the Mod group (*P* < 0.01) (**Figure 2C**).

#### General Morphology Score

Based on the above observations, the colon from each group was subjected to gross morphological scoring. Most mice from the Con group had no congestion, edema, or ulcer, and only a small part of the colon mucosa showed minor congestion. In the Mod group, the colonic mucosa was diffusely congested, consistent with edema and ulcers; some ulcers had necrotic tissues on the surface. In the Mes, TP1, and TP2 groups, the mucosa of the proximal and anal colon was moderately hyperemic and edema was evident. The degree of ulceration significantly reduced as compared with that observed for the Mod group, and colonic lesion was not obvious. The scores were significantly higher in the Mod group than in the Con group (*P* < 0.01), while those for Mes, TP1, and TP2 groups were significantly lower than the score for the Mod group (*P* < 0.05) (**Figure 2B**).

#### Hematoxylin and Eosin Staining and Histopathology Score

Colonic lesions were stained with H&E and assigned histopathological scores. The results showed that the mucosal gut from the Con group was tightly arranged, and the morphology of the intestinal gland epithelial cells was normal; the structure was normal without ulcers. The mice from the Mod group showed obvious ulceration, complete loss of mucosal intestinal glands and epithelium, and several inflammatory cells. Inflammation was observed in the submucosal and muscle layer. After drug administration, inflammatory cell infiltration reduced, intestinal mucosal epithelial cell integrity improved, and colonic mucosal damage alleviated in Mes and TP groups (**Figure 2E**). The results of histopathological scoring showed that the lesion score for the Mod group was significantly higher than that for the Con group (*P* < 0.01). In comparison with the Mod group, the TP groups showed a significant decrease in pathological scores after drug administration (*P* < 0.05); the pathological score for the TP1 group significantly decreased (*P* < 0.01) (**Figure 2D**). Thus, triptolide could improve colonic inflammation and alleviate local lesion damage in DSS-induced UC mice.

### The Effect of Triptolide on the Level of Inflammatory Factors in Dextran Sodium Sulfate-Induced Ulcerative Colitis

**Figure 3:**
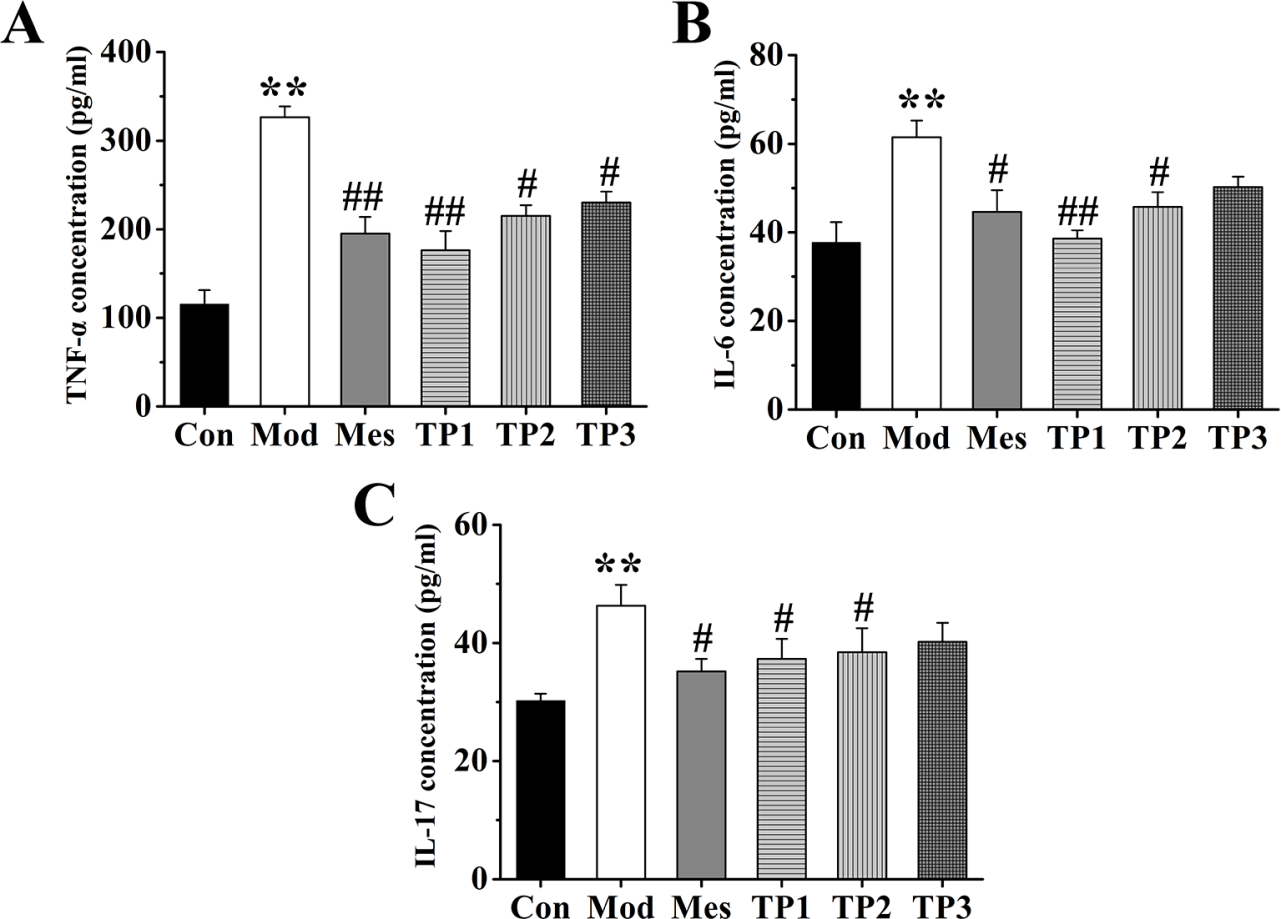
Triptolide affected the levels of inflammatory factors in the serum of mice with dextran sodium sulfate (DSS)-induced chronic colitis. **(A)** Tumor necrosis factor alpha (TNF-α) concentration. **(B)** IL-6 concentration. **(C)** Interleukin (IL)-17 concentration. After triptolide treatment, the expression of IL-6, tumor necrosis factor (TNF)-α, and IL-17 in serum samples was significantly lower than DSS-induced ulcerative colitis (UC) model mice. *P < 0.05 and **P < 0.01 *versus* Con group; ^#^ *P* < 0.05 and ^##^ *P* < 0.01 *versus* Mod group.

The inflammatory factors in the serum from each group were detected. The expression levels of IL-6, TNF-α, and IL-17 were examined and found to be the lowest in the serum of mice from the Con group; the highest levels were observed in the samples from Mod group that were significantly different from the levels detected in Con group. The expression of IL-6, TNF-α, and IL-17 in serum samples from Mes and TP groups was significantly lower than that in the samples from Mod group (*P* < 0.01). Thus, triptolide could inhibit the expression of IL-6, TNF-α, and IL-17 in the serum of UC mice (**Figure 3**).

### Alpha Diversity Analysis of Mouse Colonic Flora

**Figure 4:**
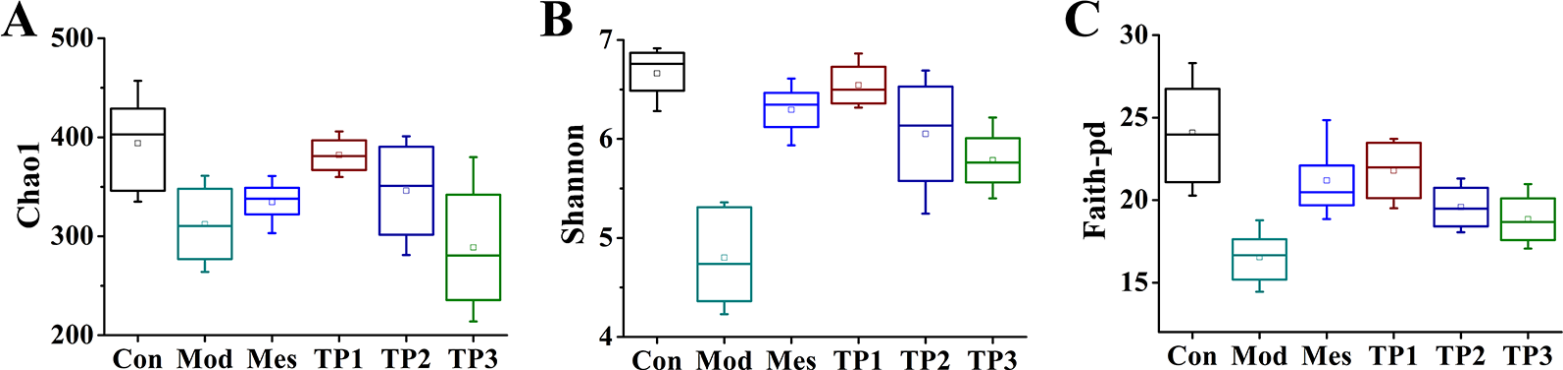
Triptolide influenced the gut microbiota of dextran sodium sulfate (DSS)-induced chronic colitis mice. **(A)** Chao1, number of operational taxonomic units (OTUs) in the sample. **(B)** Shannon, flora diversity. **(C)** Faith-pd, microbial population abundance.

Alpha diversity index is the analysis of species diversity in any sample and indicates the richness and uniformity of the species composition. In general, it is evaluated using observed OTU, Shannon, and Faith's phylogenetic diversity. The higher the index, the more complex is the diversity of the sample. Diversity analysis (alpha diversity) of a single sample could reflect the abundance and diversity of microbial communities, including statistical analysis indices, to estimate species abundance and diversity of environmental communities. The Chao index is used to calculate the abundance of flora and estimate the number of OTUs present in the sample using the Chao1 algorithm. Chao1 is commonly used in ecology to estimate the total number of species. Shannon index is used to calculate the index of diversity of the flora. The Simpson diversity index is often used to reflect the alpha diversity index. The larger the Shannon value, the higher is the diversity of the community. The Faith-pd number indicates the abundance of microbial population and includes the abundance of rare species. The results showed that the treatment with 3% DSS resulted in a decrease in the total number of gut microbiota in each group, with the lowest number of species observed in the Mod group. After treatment with triptolide, the total number of gut species was significantly recovered (**Figure 4A**). After colitis, the diversity of the gut microbiota in each group significantly reduced, but the treatment with triptolide significantly restored the diversity of gut microbiota (**Figure 4B**). The microbial population abundance was averaged after the induction of model, while the abundance of rare species increased after triptolide treatment (**Figure 4C**).

### Beta Diversity Analysis of Mouse Gut Microbiota

**Figure 5:**
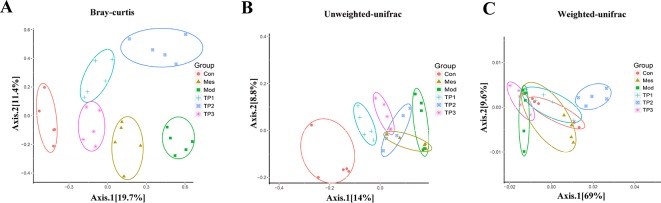
Triptolide affected the gut microbiota of dextran sodium sulfate (DSS)-induced chronic colitis mice. **(A)** Principal coordinate analysis (PCoA) based on Bray Curtis. **(B)** PCoA based on unweighted Unifrac. **(C)** PCoA based on weighted Unifrac.

Beta diversity analysis compares the composition of microbial communities between different samples. Bray Curtis, weighted Unifrac, and unweighted Unifrac distances were calculated from the OTU abundance information of the samples to assess the differences in the microbial community between different samples. Bray Curtis distance is the most commonly used indicator of the difference between ecologically reactive communities and considers only the species abundance information. The unweighted Unifrac distance is the distance between samples based on the evolutionary relationship between species systems and considers only the presence or absence of species. The weighted Unifrac distance is the distance between samples obtained after combining the abundance information of OTU with the evolutionary relationship of the system. Unweighted Unifrac distances are sensitive to rare species, while Bray Curtis and weighted Unifrac distances are more sensitive to highly abundant species. Weighted Unifrac distance, unweighted Unifrac distance, and Bray Curtis distance as beta diversity distance are indicators used to measure the coefficient of dissimilarity between two samples. The smaller the value, the smaller is the difference in species diversity between two samples. Principal coordinate analysis (PCoA) is a similarity-reduction method for principal component analysis (PCA). The most important elements and structures extracted from multi-dimensional data can extract three coordinate axes that reflect the difference between samples to the greatest extent. The difference is reflected in the three-dimensional coordinate map, which reveals the simple law in the background of complex data. We perform PCoA based on Bray Curtis distance, weighted Unifrac distance, and unweighted Unifrac distance, and selected the main coordinate combination with the largest contribution rate for graph display. The closer the sample distance, the more similar was the species composition structure of the sample. PCoA showed that the biological composition of the gut microbiota significantly changed in the mice from Mod group as compared with that in the mice from Con group. The biological composition of the gut microbiota also significantly changed for the mice after treatment with TP. The difference between the TP and Con group was reduced (**Figure 5**).

### Gut Microbiota Species Annotation and Difference Analysis

**Figure 6:**
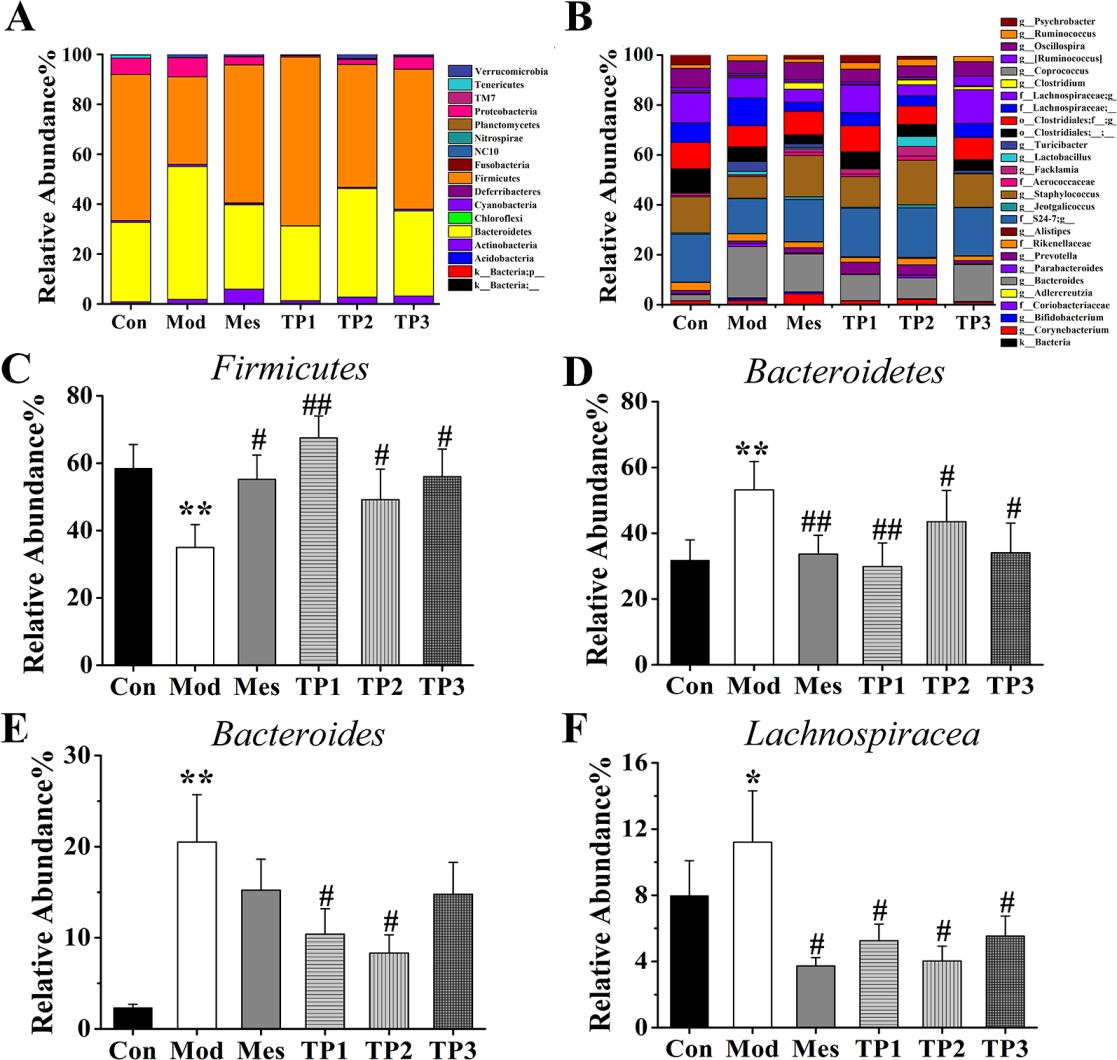
Effects of triptolide on the gut microbiota of mice treated with dextran sodium sulfate (DSS). **(A)** Rank of phylum. **(B)** Rank of genus. **(C, D)** At the phylum level, the relative abundance of *Firmicutes* was increased in TP group compared with Mod group whereas decreased the relative abundance of *Bacteroidetes*. **(E, F)** At the genus level, the relative abundances of *Bacteroides* and *Lachnospiraceae* in the TP group were lower than those in the Mod group. *P < 0.05 and **P < 0.01 *versus* Con group; ^#^ *P* < 0.05 and ^##^ *P* < 0.01 *versus* Mod group.

Based on the absolute abundance and annotation information of OTU, the ratio of the number of sequences at phylum and genus levels to the total number of sequences in each sample was statistically analyzed to evaluate differences in species composition at phylum and genus levels in each group (**Figure 6**).

The flora spectrum reflects the composition of the gut microbiota of normal mice. *Bacteroidetes* (31.76%), *Firmicutes* (58.46%), *Proteobacteria* (6.58%), and *Tenericutes* (1.24%) are the four major bacterial species. *Bacteroides*, *S24-7*, *Staphylococcus*, *Clostridiales*, *Lachnospiraceae*, *Oscillospira*, *Ruminococcus*, *Psychrobacter,* etc. are the main components of the gut microbiota of mice from the Con group. The changes in the main genus play a role in the establishment of the intestinal bacterial barrier.

The microbial spectrum of this group is reflective of the destruction in the composition of the gut microbiota by 3% DSS. After treatment with 3% DSS, the composition ratios of the following four major bacteria significantly changed: *Bacteroidetes* (53.19%), *Firmicutes* (35.01%), *Proteobacteria* (7.64%), and *Tenericutes* (0.10%). In comparison with the Con group, the Mod group showed an increase in the proportion of *Bacteroides*, *Ruminococcus*, and *Lachnospiraceae* and a decrease in the proportion of *S24-7*, *Staphylococcus*, *Clostridiales*, *Oscillospira*, and *Psychrobacter* at the genus level.

The mice from this group were treated with mesalazine for 7 days and the intestinal contents were retrieved. The flora reflected the effect of mesalazine on the gut microbiota. The major bacterial population included *Bacteroidetes* (33.67%), *Firmicutes* (55.20%), *Proteobacteria* (3.24%), and *Tenericutes* (0.18%). At the genus level, the proportion of *S24-7*, *Staphylococcus*, *Oscillospira*, *Ruminococcus*, and *Psychrobacter* was higher and that of *Bacteroides*, *Clostridiales,* and *Lachnospiraceae* was lower than that observed in the Mod group.

The mice from this group were treated with triptolide for 7 days, and the contents of the intestinal tract were retrieved. The flora reflected the effect of each dose of triptolide on the gut microbiota. In the TP1 group, the main bacteria included *Bacteroidetes* (29.87%), *Firmicutes* (67.53%), *Proteobacteria* (0.55%), and *Tenericutes* (0.30%), while *Bacteroidetes* (43.50%), *Firmicutes* (49.15%), *Proteobacteria* (2.01%), and *Tenericutes* (0.13%) represented the major population in the TP2 group. The TP3 group showed *Bacteroidetes* (34.07%), *Firmicutes* (55.99%), *Proteobacteria* (5.05%), and *Tenericutes* (0.25%) as the major population. In comparison with the Mod group, TP groups showed an increase in the proportion of *S24-7*, *Staphylococcus*, *Clostridiales*, *Oscillospira*, *Ruminococcus*, and *Psychrobacter* and a decrease in the proportion of *Bacteroides* and *Lachnospiraceae*.

### Linear Discriminant Analysis Effect Size and Functional Predictive Analyses

**Figure 7:**
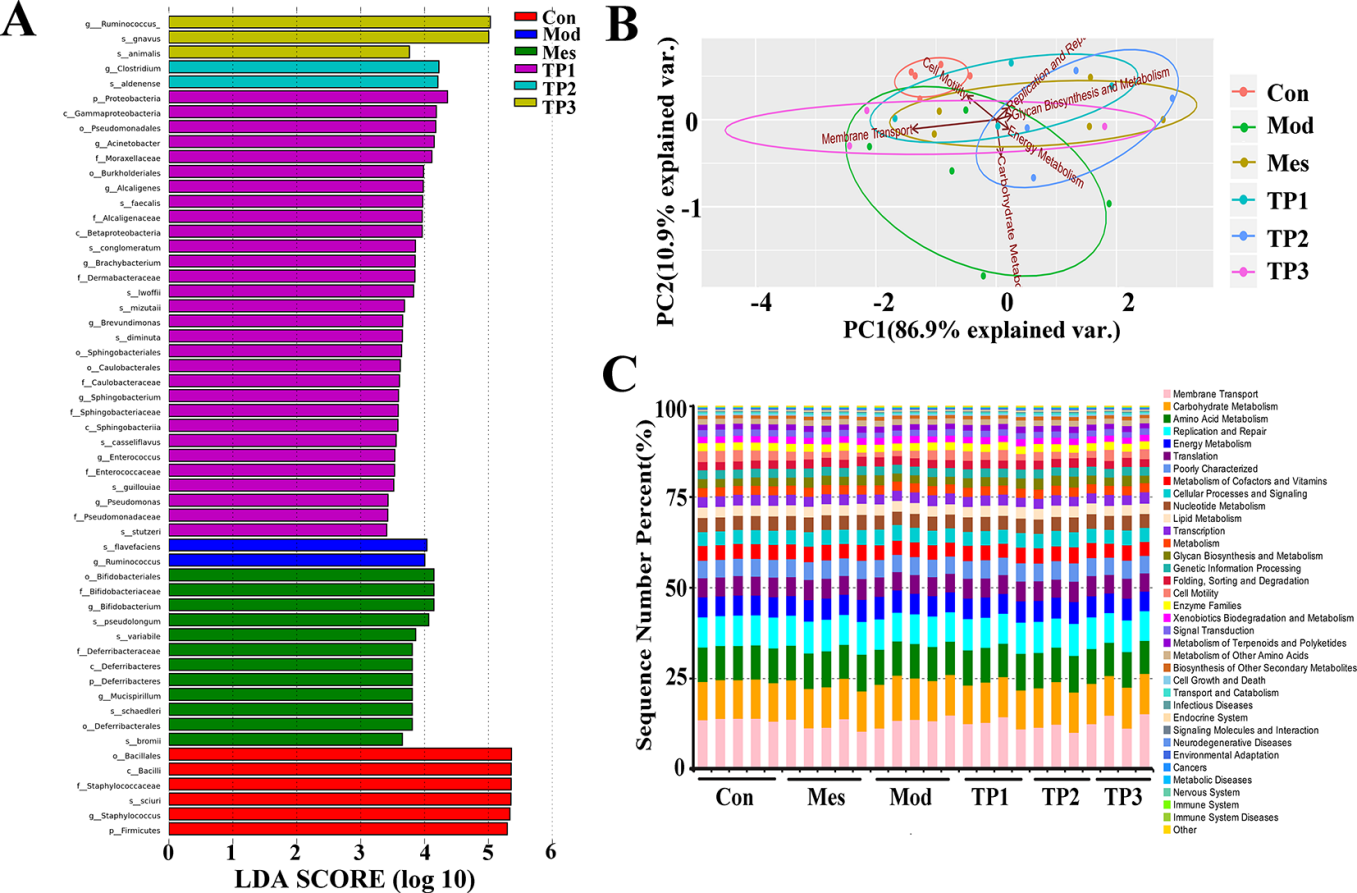
Linear discriminant analysis effect size (LEfSe) and functional predictive analyses. **(A)** Linear discriminant analysis effect size identifies the differential abundance of bacteria. **(B)** PCA results based on Kyoto Encyclopedia of Genes and Genomes (KEGG) L2 level. **(C)** Composition column chart of KEGG L2 microbial community function prediction.

We used linear discriminant analysis effect size (LEfSe) analysis to compare the difference in the abundance of gut microbiota at species level after treatment with 3% DSS. The LEfSe method is a combination of non-parametric test and linear discriminant analysis and is suitable to detect differences in bacterial abundance. The linear discriminant analysis (LDA) value is considered to be greater than 3.5 as the screening standard to determine the abundance of microorganisms in this group. LDA histogram (**Figure 7A**) was more intuitive at the species level, with the highest significance of *Firmicutes*, *Staphylococcus*, *Sciuri*, *Staphylococcaceae*, *Bacillus*, and *Bacillales* in the Con group. The most significant population in the Mod group included *Flavefaciens* and *Ruminococcus*. The Mes group had *Bifidobacteriales*, *Bifidobacteriaceae*, *Pseudolongum*, *Variabile*, and *Schaedleri* as the most significant population, while *Ruminococcus gnavus*, *Clostridium aldenense*, *Proteobacteria*, gamma *Proteobacteria*, *Pseudomonadales*, and *Acinetobacter* were detected in TP group.

PICRUSt is based on the 16S rRNA sequences of the tested bacterial genomes and infers the gene function profiles of their common ancestors and other undetected species in the Greengenes database, thereby providing a full-spectrum gene function prediction. The flora composition obtained by sequencing is mapped into a database to predict their metabolic functions. The Kyoto Encyclopedia of Genes and Genomes (KEGG) can be divided into three classification levels according to the annotation depth, consistent with the classification level of microorganisms. It shows the PCA and histograms of the resulting L2 level functional predictions (**Figures 7B, C**). The gut microbiota mainly affects membrane transport, carbohydrate metabolism, amino acid metabolism, replication and repair, and energy metabolism. In the Mod group, the metabolic process of mice changed and the metabolic rate of amino acids decreased. Moreover, the ability of replication and repair and energy metabolism decreased, but the ability of membrane transport increased. The functional prediction of the gut microbiota from TP group was similar to that of the Con group. PCA also revealed the significant difference in the functional prediction of the gut microbiota of mice from each group.

### Triptolide Fecal Transplants Reduced Dextran Sodium Sulfate-Induced Ulcerative Colitis

**Figure 8:**
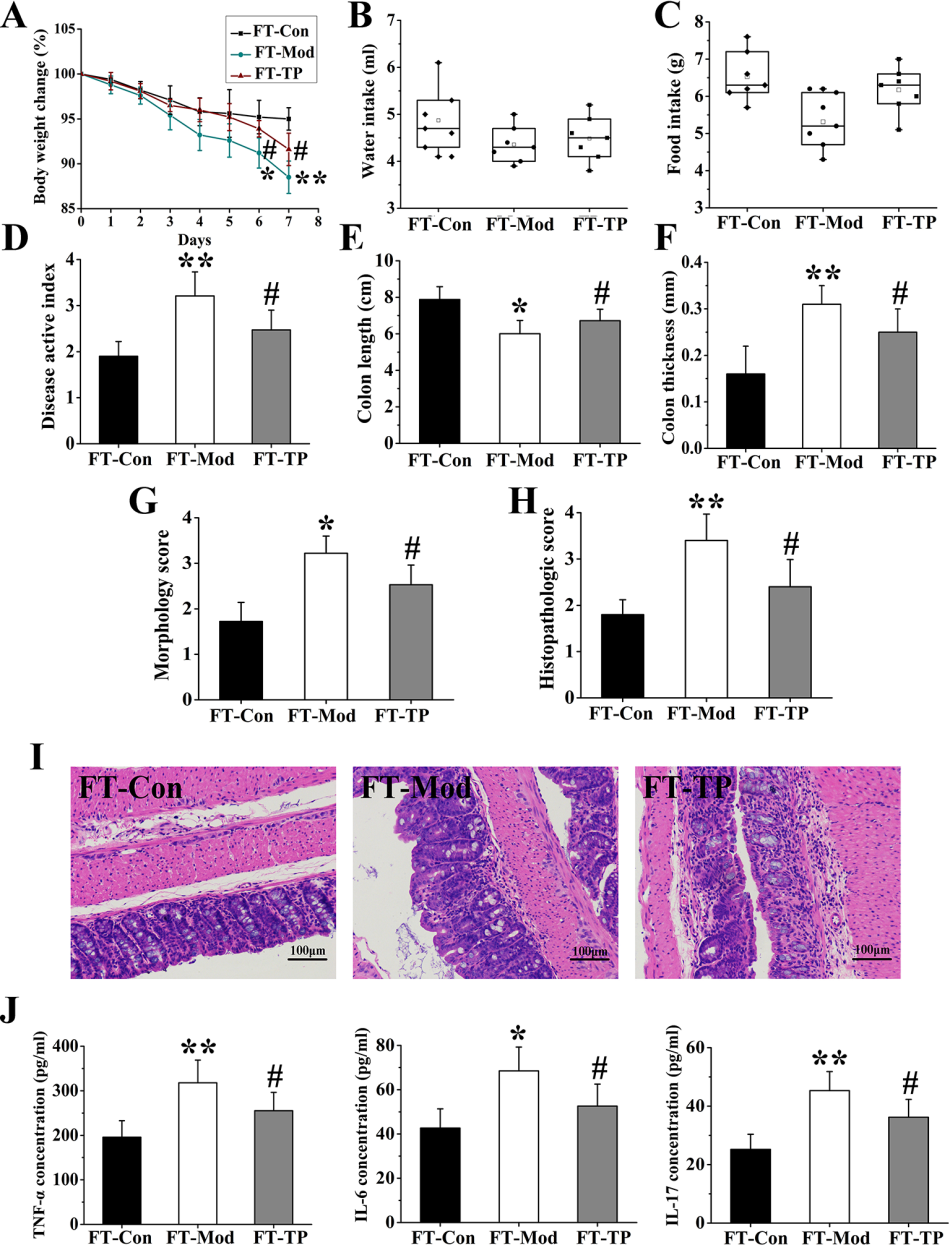
Triptolide fecal transplants reduced dextran sodium sulfate (DSS)-induced ulcerative colitis. **(A–C)** Triptolide fecal transplantation reduced body weight loss and increased food and water intake compared with FT-Mod fecal transplantation group. **(D)** Mice received triptolide fecal transplantation had a lower disease activity index (DAI) score compared with FT-Mod fecal transplantation group. **(E–G)** Mice received triptolide fecal transplantations had a significantly increased colon length, decreased colon thickness and general morphology score as compared with FT-Mod treated mice fecal transplantation group. **(H, I)** Hematoxylin and eosin (H&E) staining and histological colitis score showed that triptolide fecal transplantation reduced mucosal necrosis and inflammatory cell infiltration in colon. **(J)** The expression of interleukin (IL)-6, tumor necrosis factor (TNF)-α, and IL-17 in serum samples from mice receiving triptolide fecal transplantations group was significantly lower than FT-Mod fecal transplantation group. *P < 0.05 and **P < 0.01 *versus* FT-Con group; ^#^ *P* < 0.05 *versus* FT-Mod group.

Recent studies have shown that fecal microbiota transplantation (FMT) could treat UC by modifying the gut microbiota (Anderson et al., 2012; Kunde et al., 2013). To further confirm that triptolide could improve the UC mice by regulating the gut microbiota, gut microbiota of mice in CON, MOD, and TP groups were transferred into DSS-induced UC mice, followed by examination of related traits. The groups of FT-Con and FT-TP showed improved body weight, increased food and water intake, and reduced DAI score compared with FT-Mod group (**Figures 8A–D**). And FT-Con and FT-TP groups had a significantly increased colon length, decreased colon thickness and general morphology score as compared with FT-Mod group (*P* < 0.05) (**Figures 8E–G**). H&E staining showed that the groups of FT-Con and FT-TP had less mucosal necrosis and inflammatory cell infiltration than FT-Mod group. And it was a lower histological colitis score compared with FT-Mod group (*P* < 0.05) (**Figures 8H, I**). The expression levels of IL-6, TNF-α, and IL-17 were examined and found to be the lowest in the serum of FT-Con group; And the expression of IL-6, TNF-α, and IL-17 in serum samples from FT-TP group was significantly lower than that in the samples from FT-Mod group (*P* < 0.05) (**Figure 8J**).

Through the above experimental results, we found that the abundance of flora, the index of diversity of the flora and the abundance of microbial population and includes rare species in the Con group were the highest. After 3% DSS induction, these indicators had decreased. These may be the main reasons for the significant difference in the therapeutic effect on UC mice after fecal microbiota transplantation. After treatment with triptolide, the total number of gut species was recovered, the abundance of rare species was increased, and the microbial diversity was significantly improved. The rise of these indicators also represents the restoration of gut microbiota function. Changes in microbiota may have an effect on intestinal mucosal barrier and intestinal metabolism. And these may be factors of gut microbiota mediate triptolide efficacy after fecal microbiota transplantation.

## Discussion

The interaction between the host immune system and the gut microbiota is thought to be associated with the onset of UC and the difficulty underlying its treatment. The normal gut microbiota serves as the barrier and protects the intestinal tract. Changes in the composition of gut microbiota may affect the micro-ecology of the intestine, which in turn affects immune and metabolic functions and consequently leads to a variety of autoimmune and intestinal diseases. Therefore, the gut microbiota is thought to be involved in the occurrence and development of UC.

Nishikawa et al. used PCR restriction fragment length polymorphism (RFLP) technology to compare the number of colonies in the intestine of patients with UC and normal subjects (Nishikawa et al., 2009). These authors found that the number of restriction fragments obtained in the stool of patients with UC was significantly lower than that detected in the samples from healthy controls. Macfarlane et al. analyzed the number and composition of gut microbiota in patients with inflammation and normal subjects and found that the ratio of anaerobic to facultative anaerobic bacteria was 20:1 in normal population but changed to 2.5:1 in patients (Macfarlane et al., 2004). In addition, Andoh et al. collected fresh feces from active patients with UC and healthy subjects and analyzed the bacterial species using RFLP of bacterial 16S rRNA gene (Andoh et al., 2011). The number of clostridia in the intestinal tract of patients with UC was found to be significantly reduced, while that of *Bacteroides* significantly increased. The gut microbiota plays an important role in UC, which is characterized with a decrease in bacterial diversity, destruction of normal flora, and growth of pathogenic bacteria.

Triptolide is an epoxidized diterpene lactone compound isolated from *T. wilfordii* and serves as one of the main constituents in traditional Chinese medicine. Triptolide has a rosin skeleton and contains a unique triepoxy structure and an α, β-unsaturated five-actactone ring. Pharmacological and clinical tests have demonstrated its immunosuppressive, anti-inflammatory, and other biological activities (Banerjee et al., 2013; Lin et al., 2001; Wu et al., 2013; Zheng et al., 2013). Triptolide exerts positive effects on UC (Wei et al., 2008; Zhang and Chen, 2017). Here, we used a DSS-induced UC mouse model and recorded general signs, DAI, and colon length, thickness, lesion gross score, and histopathology score along with the analysis of serum levels of TNF-α, IL-6, and IL-17. The results showed that triptolide exerted therapeutic effects in mice with UC.

Fecal transplantation is a new method to study the relationship between herbs and microbiota (Chang et al., 2017; Cui et al., 2018). In our study, fecal transplantation was performed to assess the role of triptolide in the treatment of gut microbiota during UC. Our results revealed that fecal transplantation from triptolide-treated mice could relieve UC.

The immunosuppressive effects of triptolide are closely related to the gut microbiota. **Figure S1** shows the triptolide affected the infiltration of lymphocytes in colon tissues. Studies have shown that the imbalance between Th1/Th2 and Th17/Treg cell population results in changes in expression levels of pro-inflammatory and anti-inflammatory factors, thereby causing lymphocyte infiltration and B cell activation (Bliddal et al., 2017; Shao et al., 2018). This phenomenon plays a crucial role in the pathogenesis of UC. The gut microbiota regulates the population of Th17 cells, which play an important role in resisting pathogens and regulating autoimmunity. Experiments have shown that the gut microbiota can regulate the differentiation of Th17 cells and the functions of Treg cells (Duan and Kasper, 2011; Kim et al., 2016). Treg cells control inflammatory responses and maintain autoimmune tolerance through a subset of T cells, which affect autoimmune reactivity *in vivo* (Atarashi et al., 2011; Smith et al., 2013). After triptolide treatment, the recovery rate of gut microbiota accelerated. The population of *Bacteroidetes* decreased while that of *Firmicutes* increased. In addition, the population of *Bacteroides* and *Lachnospiraceae* decreased, suggesting that the microbiota of UC mice may recover after triptolide treatment. The recovery in the flora may improve the structure of the gut microbiota.

In summary, triptolide exerts promising therapeutic effects in UC mice, and could improve the structure of the gut microbiota by accelerating the recovery of bacterial diversity. This study provides a reference for the rational use of triptolide for the treatment of UC.

## Data Availability Statement

The datasets generated for this study can be found in the Sequence Read Archive, using the accession number PRJNA577811.

## Ethics Statement

The animal study was reviewed and approved by the Animal Ethics Committee of Capital Medical University.

## Author Contributions

HW and CN performed the investigation, analyzed the data and wrote the paper. QR and ZJ designed the study and amended the paper. GC and XH helped in the execution of research.

## Conflict of Interest

The authors declare that the research was conducted in the absence of any commercial or financial relationships that could be construed as a potential conflict of interest.
