# Erratum: Effect of Triptolide on Dextran Sodium Sulfate-Induced Ulcerative Colitis and Gut Microbiota in Mice

**DOI:** 10.3389/fphar.2020.00319

**Published:** 2020-03-10

**Authors:** Frontiers Production Office

**Affiliations:** Frontiers Media SA, Lausanne, Switzerland

**Keywords:** ulcerative colitis, triptolide, gut microbiota, high-throughput sequencing, mice model

Due to a production error, affiliation 1 was incorrect. The correct affiliation is “Beijing Friendship Hospital, Capital Medical University, Beijing, China”.

The publisher apologizes for this mistake. The original version of this article has been updated.

