# Supplementary material for: Effect of Triptolide on Dextran Sodium Sulfate-Induced Ulcerative Colitis and Gut Microbiota in Mice
Source: Front Pharmacol. 2020 Jan 29;10:1652. doi: 10.3389/fphar.2019.01652 (PMC7000629; doi:10.3389/fphar.2019.01652)
Supplement: Supplementary file 1 [file DataSheet_1.docx]

***Supplementary materials for*：**

**Effect of triptolide on DSS-induced ulcerative colitis and gut microbiota in mice**

Hao Wu^1‡^, Quan Rao ^1,2‡^, Guang-Chao Ma^1^, Xiao-Hong Yu^1^, Cong-En Zhang ^1*^, Zhi-Jie Ma^1*^

*^1^ Beijing Friendship Hospital, Capital Medical University, Beijing, PR China.*

*^2^Department of General Surgery, Beijing Friendship Hospital, Capital Medical University, Beijing, PR China.*

Correspondence:

Cong-En Zhang and Zhi-Jie Ma

Beijing Friendship Hospital, Capital Medical University, 95 Yong-an Road, Xi-Cheng District, Beijing 100050, China.

Fax: +86 63138748; Fax: +86 63138562

E-mail: zce820@163.com; [13811647091@163.com](mailto:13811647091@163.com)

‡ These authors contributed equally to this work.

* To whom correspondence should be addressed.

**1. Immunohistochemistry**

**
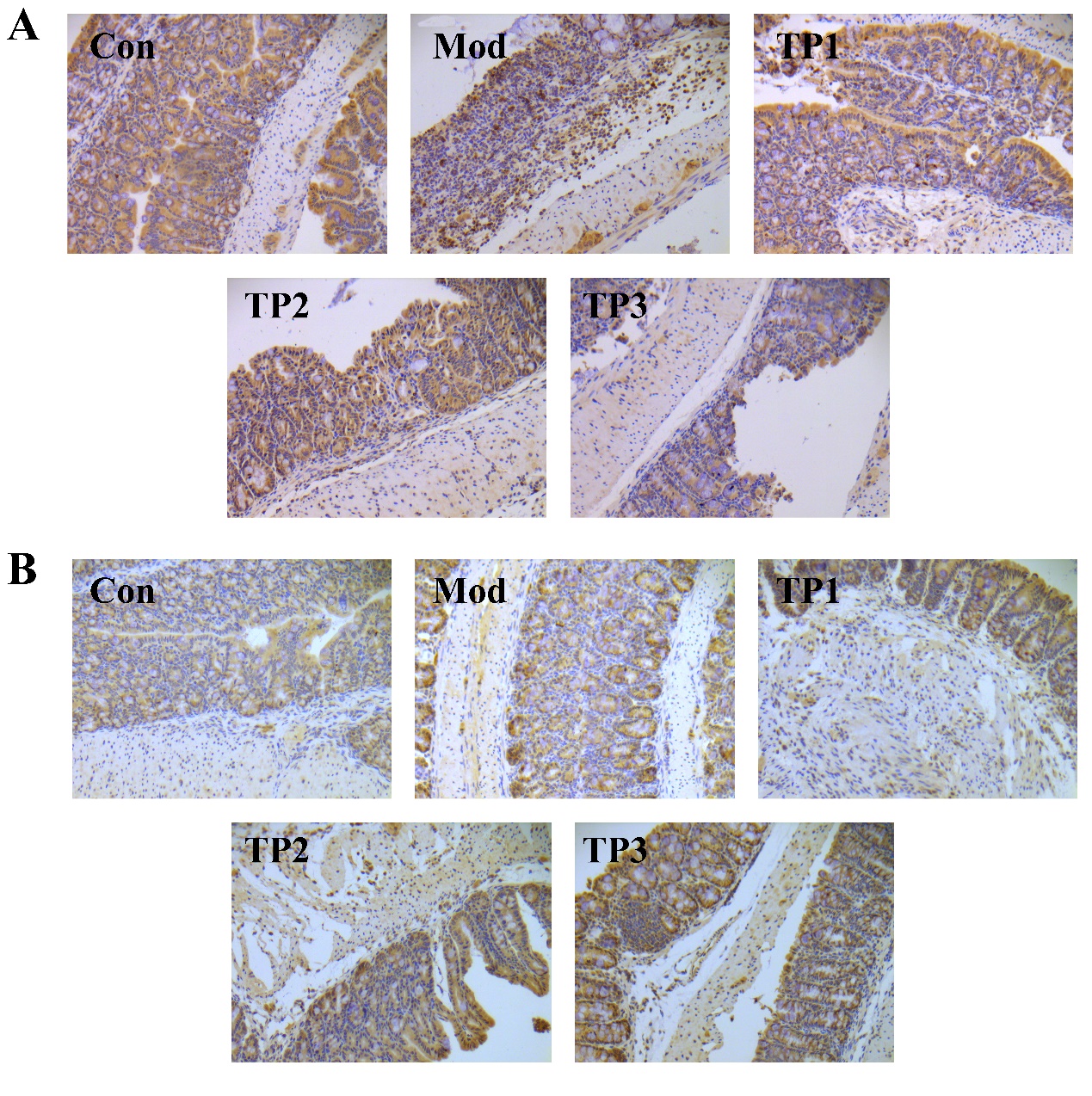
**The intestinal tissue of the UC model was obtained for immunohistochemical staining, and the expression of CD3 and FoxP3 was observed to detect changes in T cell and regulatory T cell populations in the lesions and to analyze the effects of triptolide treatment. As a result, we found a significant increase in T lymphocyte infiltration in the colon tissues of mice with UC. After treatment with triptolide, T cell infiltration reduced and inhibitory T cell population increased **(Figure S1)**. Thus, triptolide could ameliorate the infiltration of inflammatory cells in the diseased intestinal tissues and transform the surrounding immune microenvironment from immune activation to immune tolerance. This transformation is very important for the treatment and prognosis of UC.

**Figure S1:** Triptolide affected the infiltration of lymphocytes in colon tissues. (A) CD3 staining (200×). (B) FoxP3 staining (200×).
